# Corrosion Inhibition Using Harmal Leaf Extract as an Eco-Friendly Corrosion Inhibitor

**DOI:** 10.3390/molecules26227024

**Published:** 2021-11-20

**Authors:** Nasreen Al Otaibi, Hassan H. Hammud

**Affiliations:** Department of Chemistry, College of Science, King Faisal University, P.O. Box 400, Al-Ahsa 31982, Saudi Arabia

**Keywords:** Harmal leaves, anticorrosion, equivalent electrical circuit, EIS, Tafel

## Abstract

Extract of natural plants is one of the most important metallic corrosion inhibitors. They are readily available, nontoxic, environmentally friendly, biodegradable, highly efficient, and renewable. The present project focuses on the corrosion inhibition effects of Peganum Harmala leaf extract. The equivalent circuit with two time constants with film and charge transfer components gave the best fitting of impedance data. Extraction of active species by sonication proved to be an effective new method to extract the inhibitors. High percent inhibition efficacy IE% of 98% for 283.4 ppm solutions was attained using impedance spectroscopy EIS measurements. The values of charge transfer *R*_ct_ increases while the double layer capacitance *C*_dl_ values decrease with increasing Harmal extract concentration. This indicates the formation of protective film. The polarization curves show that the Harmal extract acts as a cathodic-type inhibitor. It is found that the adsorption of Harmal molecules onto the steel surface followed Langmuir isotherm. Fourier-transform infrared spectroscopy FTIR was used to determine the electron-rich functional groups in Harmal extract, which contribute to corrosion inhibition effect. Scanning electron microscopy SEM measurement of a steel surface clearly proves the anticorrosion effect of Harmal leaves.

## 1. Introduction

Inhibition of corrosion of metals represents a major challenge in the industry. The main concern is to retard the extensive corrosion of different parts of oil production plants, tubing, and pipelines from the wellhead equipments. Corrosion is caused by the contact of metal surface with dissolved carbon dioxide, hydrogen sulfide, as well as salts [1]. Metallic corrosion causes huge yearly losses in the United States [2]. Inhibition of corrosion by adsorption inhibitors has been applied in different technologies such as acid pickling and descaling, petrochemicals, and storage of chemicals [3,4].

Many inorganic compounds such as chromium and manganese organic complexes were used as corrosion inhibitors in different media [5,6,7,8,9]. These low cost complexes were able to decrease efficiently the corrosion of different metals, such as Fe, Al, and Cu [9,10]. However, their use as corrosion inhibitors leads to environmental pollution. This makes them less applicable [10,11].

The world becomes much aware of the environmental problems and the toxic effects of the used chemicals in different industries. Nowadays there is growing attention towards eco-friendly corrosion inhibitions “green corrosion inhibitors”. There are several advantages of these naturally obtained inhibitors. They are readily available, environmentally friendly, biodegradable, efficient, and renewable [12,13,14,15,16].

Those eco-friendly inhibitors can be easily extracted from plants. The next important step is to separate, identify different species, and correlate them to the inhibition effectiveness. Moreover, the study of the inhibition effects of the extracted species alone or in combination was carried out to investigate the possibility of synergism or antagonism that ensures an optimum level of inhibition [17,18,19,20,21]. The organic extracts from plant leaves, barks, seeds, fruits, and roots act as green corrosion inhibitors in different aggressive media. The extracts containing nitrogen, sulfur and oxygen functional groups exhibit good inhibition. Because, these groups having free electrons can bind to partially dissolved metal ions, retarding further dissolution and corrosion [22,23,24,25].

Recently several ecofriendly corrosion inhibitors were studied. Corrosion of mild steel in HCl solution was investigated using extracts of leaves as green inhibitors: the extract of Salvia leaves acted as anticorrosion on stainless steel using polarization studies. The efficiency was due to the presence of phenolic components [26]. The inhibition of carbon steel corrosion was investigated using Phyllanthus amarus leaf extract [27]. The adsorption of plant extract followed Langmuir isotherm. Also, polarization curves indicated that the plant extract act as an anodic inhibitor and cathodic inhibitor. Alkaloids extract of Geissospermum leave was studied as an inhibitor for carbon C-steel corrosion using electrochemical methods, Scanning electron microscopy SEM and Energy-dispersive X-ray spectroscopy EDX [28]. The results indicated that plant extract has high inhibition efficiency IE% of 92%. Also, Acalypha torta leaves extracts were used to inhibit corrosion of mild steel. Weight loss, electrochemical procedure, and UV-Visible spectroscopy was used to assess the inhibition efficiency. The reported inhibition efficiency was nearly 90% at 1000 ppm extract solution [29].

As well as extracts from Aloe Vera leaves were studied in 1 M H_2_SO_4_ solution. Electrochemical and SEM techniques were applied to study the inhibition effect of the extract on corrosion. The results indicated that plant extract acts as a mixed type inhibitor for mild steel with IE% of 98% at 30% *v*/*v* concentration [30]. Extracts of Citrus aurantium leaves have been employed as an eco-friendly corrosion inhibitor for mild steel in aqueous acid media. The results indicated that adsorption of molecular extract followed Langmuir isotherm [31].

Furthermore, many types of green corrosion inhibitors have been evidenced such as Cassia tora. They showed highly effective corrosion inhibition in NaCl media [32]. Also extract of Ricinus was studied to reduce the mild steel corrosion in aqueous solution. The extract showed IE% of 84% at 300 ppm concentration [33]. Neem and African star apple leaf extracts were used as green corrosion inhibitors for mild steel in NaCl and polluted seawater media [34,35]. The corrosion inhibition of steel in neutral chloride solution using Nicotiana leaves extract was studied using electrochemical techniques [36]. The investigated results showed that Nicotiana extract acts as a mixed type inhibitor. The effect of Ficus carica leaf extract on steel anticorrosion in petroleum solution was investigated [37]. Results indicate that the extract show percent inhibition efficiency IE% of 70–80% and act as cathodic and anodic inhibitors. The adsorption of extract caused corrosion inhibition and obeyed Langmuir isotherm model.

**Figure 1:**
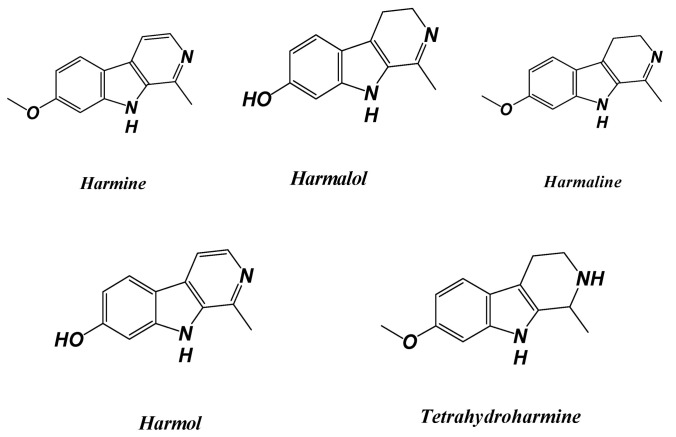
The chemical structure of the major constituents of Harmal extract.

In the current project, we studied the corrosion inhibition of carbon steel in 0.25 M H_2_SO_4_ using plant Harmal extract that grows in the desert of Saudi Arabia. The constituents of Harmal extract are shown in Figure 1, [38]. Harmal leaves extract unlike other synthesized chemicals are expected to be biodegradable. They can be decomposed by bacteria or other living organisms and thereby avoiding pollution. Harmal leaves extract contains flavonoids, alkaloids, and anthraquinones. Flavonoids are beneficial to human body due to their anti-oxidant activity. Anthraquinones are antibacterial and antiviral. Alkaloids such as harmine and harmol are biologically active. They are cytotoxic to cancer cells and act as an antiviral and antioxidant. However, they are toxic at high doses, causing hallucination [38,39]. *P. harmal* has a wide range of pharmacological effects. However, there has been several reports of intoxications due to ingestion of specific quantity of *P. harmal* seeds. Thus, care should be taken by clinicians regarding usage of this plant [40].

This project study the use of Harmal plant extracts as green corrosion inhibitor. The extract contains electron rich nitrogen and oxygen functional group that can bind to electron poor metal ion present on the exposed metallic surface. Further study is underway to make the extract commercially viable model. This project use impedance and potentiodynamic measurement in order to determine the efficiency of inhibition. The determination of corrosion properties can be also useful in energy production [41,42].

## 2. Experimental

### 2.1. Materials

Chemicals: Ethanol absolute (≥99.8%), Honeywell-France; Sulfuric acid (95–98%), Scharlau, Spain.

#### 2.1.1. Extraction of Harmal Leaves

A new extraction method “sonication” was used in this research. This method has proven effective in shortening the time. Compared to classical method where the plant is usually soaked for up to days, and extracted by using the Soxhlet apparatus several times. This ultrasound-assisted extraction not only reduce extraction times but also extractant volumes [43].

Harmal leaves were collected from Alhasa in the east regain of Saudi Arabia. The collected leaves were dried in the shad, and then ground to powder. 100 g powder of Harmal leaves were added to 100 mL ethanol in the bottom flask, which was then continuously sonicated for 1 h at 80 kHz using a Power Sonic405. The mixture was then filtered, and dried using a rotary evaporator. 1 g of green powder was obtained (1% yield).

The prepared dried Harmal extract is readily soluble in water, methanol, and ethanol. A stock solution of 10,000 ppm Harmal extract was prepared by dissolving 250 mg extract in 25 mL deionized water. In the inhibition of C-steel experiments, aliquots from this stock solution were used to prepare diluted Harmal solutions of concentrations (0, 20.79, 41.49, 62.11, 82.65, 204.1, 283.4, 625.0 and 826.5 ppm) in H_2_SO_4_ (0.25 M) medium.

#### 2.1.2. Preparation of the Specimens

The carbon steel rod with the following chemical composition % weight (wt%) C, 0.164; S, 0.001; Mn, 0.710; P, 0.0005; Si, 0.26, Ni, 0.123; Cr, 0.041; balance Fe, was used as a working electrode in electrochemical study. The exposed area of cylindrical carbon C- steel of cylindrical shape was (0.8 cm^2^). The working electrode was buffed with a series of silicon carbide papers from 600 to 1200 before the experiments. Each electrode was immersed in 0.25 M H_2_SO_4_ solution containing 20.79–826.50 ppm of the Harmal extract. C-steel plates with dimensions of 1 cm × 1 cm × 0.1 cm and chemical composition (wt%) of C 2.41%, Mn 0.69%, S 0.27%, (the balance was Fe) were immersed in Harmal extract solutions. The morphology of surfaces of plates was monitored by SEM microscope in order to study the effect of anticorrosion.

### 2.2. Methods

#### 2.2.1. Characterization of Harmal Leaves

FTIR analysis was used to determine the most important functional groups in the dried Harmal leaves and their extract. Infrared (IR) absorption bands were recorded using Agilent Technologies Cary 630 FTIR. UV-visible spectra was recorded using Shimadzu UV-1800 UV/Visible Scanning Spectrophotometer.

#### 2.2.2. Scanning Electron Microscopy

The surface morphology of C-steel specimens immersed for 3 h in 0.25 M H_2_SO_4_ or 0.25 M H_2_SO_4_ + Harmal extract respectively, were studied using a FEI Quanta FEC 250 Scanning electron microscopy (SEM) microscope. The specimens were immersed in the test solutions for 2 h before analysis at room temperature.

#### 2.2.3. Electrochemical Studies

The electrochemical study was performed at room temperature. Electrochemical impedance study (EIS) and Potentiodynamic polarization study (PDP) measurements were carried out using (Gamry, reference 600 Potentiostat/Galvanostat/ZRA, Warminster, PA, USA) and Gamry software v7.07. The cell used consisted of a platinum wire auxiliary electrode (PtE), and a saturated calomel reference electrode (SCE) electrode. C-steel rod with area (0.5027 cm^2^) was used as working electrode. Before the start of the experiment, the steady-state open circuit potential *E*_OCP_ was measured by immersing the electrode in the corrosion solution for 15 min with the indication 10 mV disturbance capacity.

#### 2.2.4. Electrochemical Impedance Study (EIS)

EIS was measured at frequency range 0.1–100,000 Hz with a signal amplitude perturbation of 10 mV around the corrosion potential. Before the start of the experiment, the electrode is immersed in the corrosion solution with or without Harmal extract at different concentrations till reaching a steady state. The obtained impedance results were represented as Nyquist plot and Bode plot. The following Equation (1) is used to calculate the inhibition efficiency (IE%) from Nyquist plot [44].

(1) $$\mathrm{IE}\%=\left(1-\frac{R\mathrm{ct}\left(0\right)}{R\mathrm{ct}\left(i\right)}\right)\times 100$$

where *R*ct(0) is the charge transfer resistance of C-steel without Harmal extract. *R*ct(i) is the charge transfer resistance of C-steel with Harmal extract. Also, the surface coverage θ were calculated using Equation (2):

(2) $$\theta =1-\frac{R\mathrm{ct}\left(0\right)}{R\mathrm{ct}\left(i\right)}\;$$

#### 2.2.5. Potentiodynamic Polarization Study (PDP)

Measurements of polarization curves were obtained by automatically polarizing the working electrode from 500 mV versus the rest potential with a scanning rate of 5 mV/s. Corrosion current density (*i*_corr_) were obtained from the extrapolation of the points to corrosion potential Ecorr. Equation (3) below shows the calculation of IE(%) from the *i*_corr_ values [45].

(3) $$\mathrm{IE}\%=\left(1-\frac{i\mathrm{corr}\left(i\right)}{i\mathrm{corr}\left(0\right)}\right)\times 100$$

where *i*_corr(i)_ and *i*_corr(0)_ are the corrosion current density of C-steel with and without Harmal extract respectively. Also, θ was calculated using the following Equation (4):

(4) $$\theta =1-\frac{i\mathrm{corr}\left(i\right)}{i\mathrm{corr}\left(0\right)}$$

## 3. Results and Discussion

### 3.1. FT-IR and UV-Visible Spectroscopy of Harmal Extract

#### 3.1.1. FTIR Spectra

**Figure 2:**
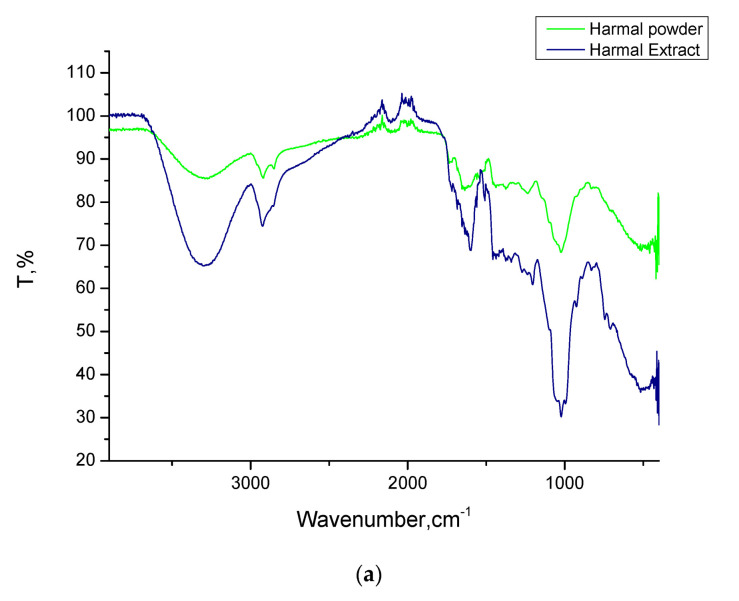

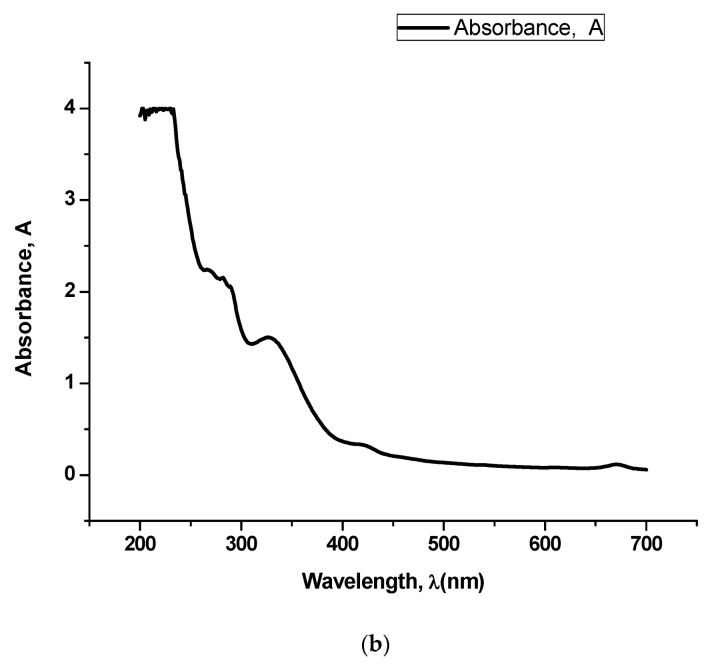
(**a**) FTIR spectra of Harmal leaves before and after extraction. (**b**) UV-visible spectra of Harmal extract aqueous solution.

Figure 1 shows the chemical structures of the major constituents of the tested Peganum Harmala extract [38]. The structures contain various electron-rich functional groups such as amine, imine fused pyrrole, hydroxyl, ether and aryl groups. These constituents with functional groups containing oxygen and nitrogen atoms can donate lone pair electrons and bind to the metal ions. Therefore, they can be particularly useful as inhibitors for metal corrosion [46]. FTIR analysis of dried Harmal leaves and extract was undertaken to further confirm the presence of these groups in the materials, Figure 2a. The stretching bands at 3294.12 cm^−1^ is due to O-H and N-H stretch, while the bands at 2939.01 and 2832.78 cm^−1^ are related to the C-H stretching frequency. The broad medium band centered at around 1593.44 cm^−1^ is related to C=N stretch and aromatic rings C=C stretch. It is shifted to lower wavenumbers in the extract compared to the dried Harmal leaves. The C-N stretch and C-O stretch can be assigned to the strong peak at 1017.56 cm^−1^. The FT-IR results indicated that the Harmal extract contains nitrogen and oxygen functional groups that can act as adsorption centers on the surface of iron steel. Comparison of the FTIR results of dried Harmal and its extract indicates that the extraction method is effective.

#### 3.1.2. UV-Visible Spectra

In the UV-Vis spectra the presence of one or more peaks in the 200 to 400 nm region is an indication of the presence of unsaturated groups and functional groups with heteroatoms such as sulfur, nitrogen, and oxygen. Figure 2b shows UV-Vis the spectrum for Harmal extract. Peak at the positions 334 nm reveals the presence of alkaloids, [47]. These results are in agreement with the FTIR results.

It is out of the scope of the present work to identify the individual species present in the extract of Harmal leaves. But one can refer to Fahmy et al. work. Where, ultraperformance liquid chromatography−electrospray ionization−tandem mass spectrometry (UPLC/ESI-MS) and reversed-phase high-performance liquid chromatography (RP-HPLC) were used to identify 15 alkaloids present in Harmal seed extracts, like harmine, harmol, etc. [39].

### 3.2. Electrochemical Impedance Spectroscopy Measurements (EIS)

**Figure 3:**
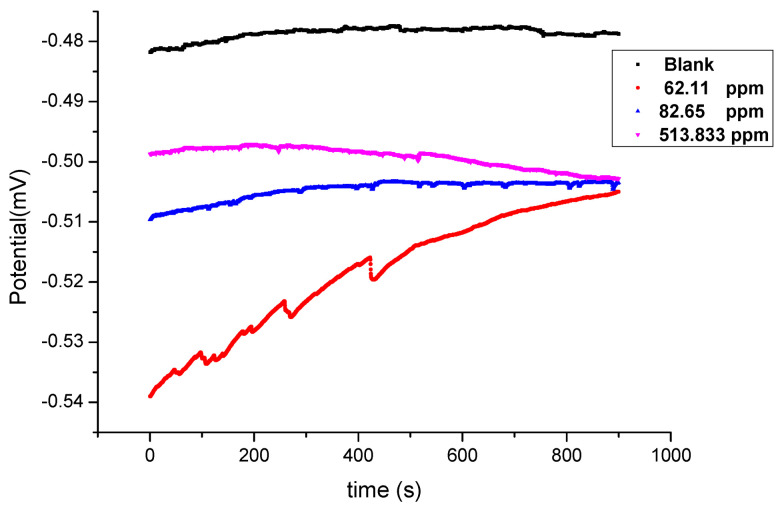
The open circuit potential E_OCP_ plot versus time (s) of C-steel with and without the addition of Harmal extract in 0.25 M H_2_SO_4_ medium.

The open-circuit potential, *E*_OCP_ was applied 15 min before electrochemical impedance study (EIS) experiments. This method studies the amount of current flow and the value of resistance in presence and absence of the Harmal extract. Stable values of *E*_OCP_ of C-steel were achieved after 900 s for all samples in 0.25 M H_2_SO_4_ medium with and without the addition of Harmal extract. The obtained results are shown in Figure 3. The *E*_OCP_ values are shifted in the negative direction as the Harmal extract is added. This behavior is related to the adsorption of inhibitor molecules on active sites on the surface of C-steel [48,49,50].

**Figure 4:**
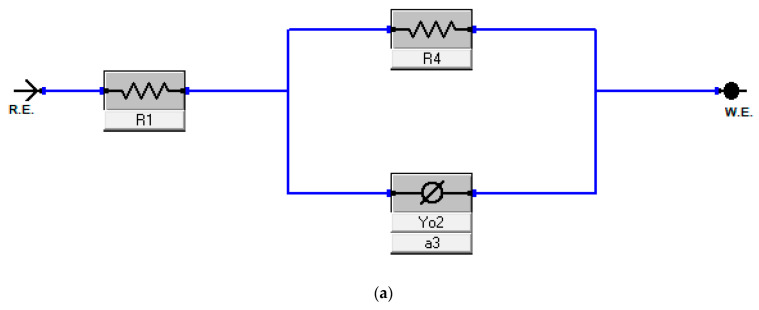

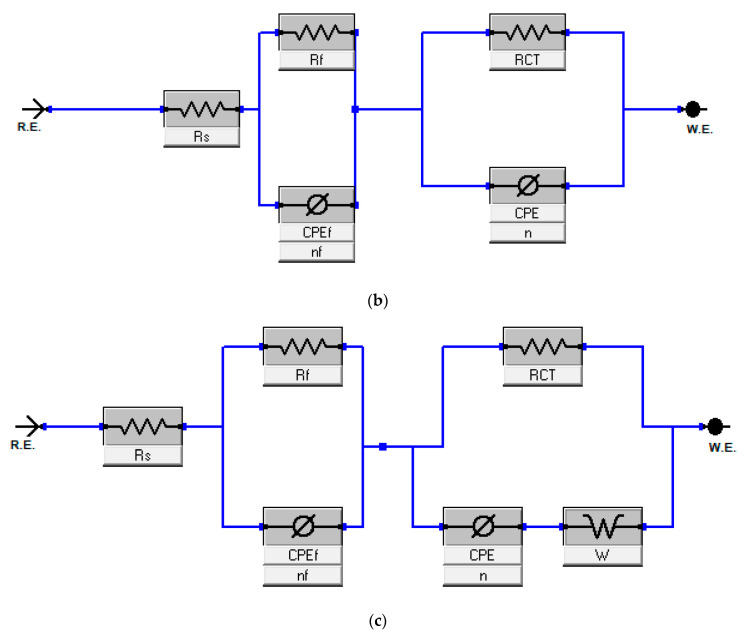
The electrical equivalent circuit (EEC) used to fit the impedance plots data (**a**–**c**) for models A, B and C respectively. (**a**) Model A. (**b**) Model B. (**c**) Model C.

**Figure 5:**
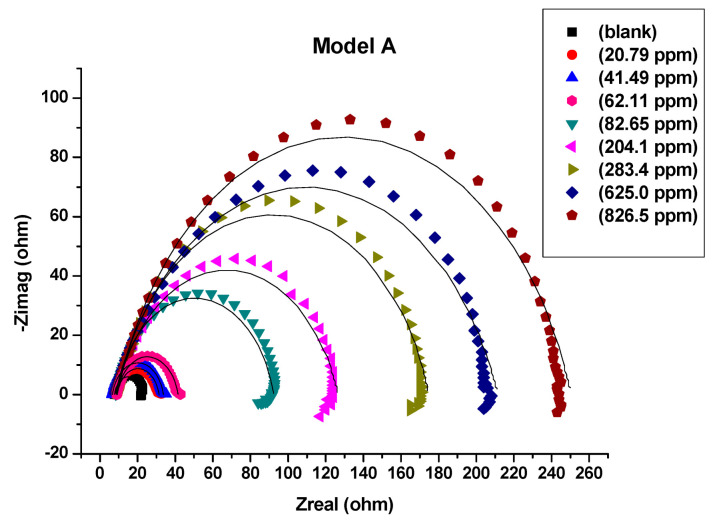
Nyquist plots in 0.25 M H_2_SO_4_, fitted by equivalent circuit model A for all concentrations of Harmal extract (0, 20.79, 41.49, 62.11, 82.65, 204.1, 283.4, 625.0 and 826.5 ppm).

**Figure 6:**
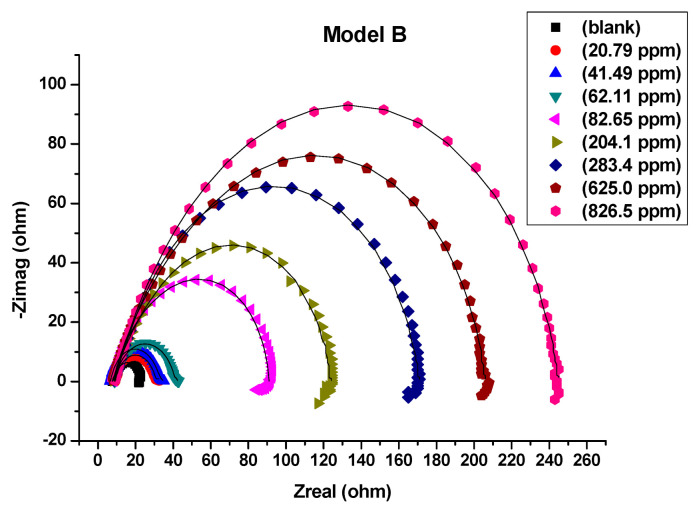
Nyquist plots in 0.25 M H_2_SO_4_, fitted by equivalent circuit model B for all concentrations of Harmal extract (0, 20.79, 41.49, 62.11, 82.65, 204.1, 283.4, 625.0 and 826.5 ppm).

**Figure 7:**
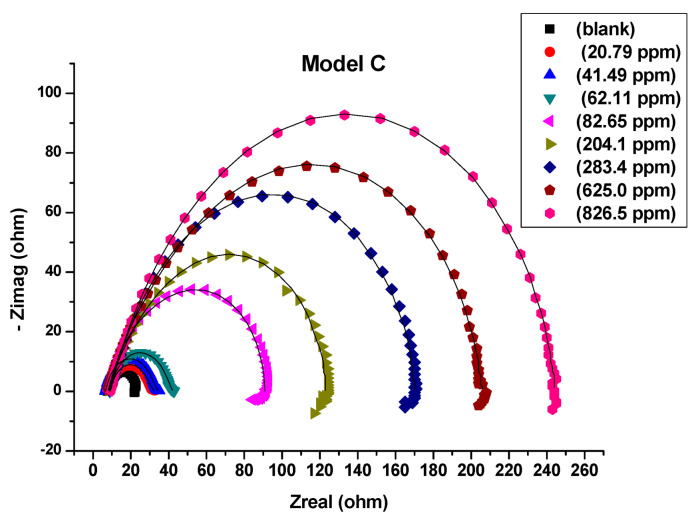
Nyquist plots in 0.25 M H_2_SO_4_, fitted by equivalent circuit model C for all concentrations of Harmal extract (0, 20.79, 41.49, 62.11, 82.65, 204.1, 283.4, 625.0 and 826.5 ppm).

All measurements of electrochemical impedance were fitted against equivalent electrical circuit (EEC) models A, B and C that were shown in Figure 4a–c. The Nyquist plots (Figure 5, Figure 6 and Figure 7) show that the presence of Harmal extract does not change the impedance diagram “semicircle”. This behavior indicates that the charge transfer process mainly controls the corrosion of C-steel. It is also noted that the addition of the Harmal extract leads to an increase in the diameter of the Nyquist plot with an increase in the inhibitor concentration. This can be due to the increase in the number of adsorbed Harmal molecules on the surface of C-steel with increasing Harmal extract concentration. In general, a deviation from a semicircle indicates some heterogeneity or roughness of the surface of the C-steel [51]. 

The EEC model A with only one time constant (The Randle’s CPE equivalent circuit) is used in order to fit the impedance data, Figure 5. The obtained electrochemical parameters with the protection efficiency are recorded in Table 1. The resistance of electrolyte (*R*_s_) is shorted by a constant phase element (CPE) that is in parallel to the charge transfer resistance (*R*_ct_). (*R*_s_) describes the ohmic resistance while (*R*_ct_) represents the inhibitor’s resistance towards oxidation of the metal surface by retarding the electrolytes reaching the coating/metal interface, and it is inversely proportional to the corrosion rate [52]. Pure double layer capacitor (*C*_dl_) is replaced by a constant phase element (CPE) to provide a more precise fit of the Nyquist plot [53]. The Nyquist plot reveals that increasing the concentration of inhibitor causes an increase in both the diameter of the semicircles and the *R*ct values, hence an increase in the corrosion inhibition efficiency. From the ohmic law where *V* = *iR*, the higher the resistance value (*R*_ct_), the lower the electrical current (*i*) flow, the lower the number of electrons transferred across the metal surface. Thus, oxidation of iron is inhibited [52,54]. However, the impedance data at high inhibitor concentration was not well fitted by Randle’s CPE equivalent circuit, where the fitting curve is not in agreement with the experimental data in the intermediate and low frequencies.

The surface coverage θ increases from 0.340 at 20.79 ppm of extract to 0.912 at the optimum concentration 283.4 ppm. At low inhibitor concentration the inhibitor is selective and bind specifically to active surface sites that would be most vulnerable to corrosion initiation. But as the inhibitor concentration increases to an optimum value, a protective layer form with more coverage area is achieved. Further increase in concentration does not improve inhibition beyond the optimum value.

Thus, equivalent electrical circuit model B with two time constants was used, (Figure 6). It gave a better fitting of impedance data for all range of concentration including the blank solution, Table 2. In general, each electrochemical process in the circuit should be represented by a separate distinct semicircle in the Nyquist plot. But when their capacitive nature has short time to relax during the change in the boundary conditions, overlapped arcs are observed in the EIS. Also, due to the high frequency limit, the first semicircle is difficult to see [6,55,56]. This result indicates that the simple prediction of the number of the time constant of the impedance spectrum from the number of capacitive loops in Nyquist plots is inadequate [57]. The fitted model B consists of two circuits connected in series and contains solution resistance (*R*_s_), film resistance (*R*_f_), charge-transfer resistance (*R*_ct_), constant phase element of the film (CPE_f_) and of the double layer (CPE_dl_). The double layer capacitance (*C*_dl_) can be also calculated from the values of *R*_ct_, the impedance of CPE_dl_ (Z_CPE_), and the exponents of CPE_dl_ (n) [58]. *R*_f_ or coating resistance (*R*_c_) is often used for an intact film/coat which act as an isolation layer and provide a good protective performance [59].

The fitting of electrochemical data of different Harmal extract concentrations are exhibited in Table 2. The polarization resistance *R*_p_ (*R*_p_ = *R*_ct_ + *R*_f_) values always keeps higher with increasing concentrations of Harmal extract, which means that it can provide effective corrosion inhibition. It reveals that increasing the concentration results in a more compact passivation film to isolate the iron from dissolution by sulfuric acid ions [58,60].

EEC model C was applied, which added a diffusion component to model B to fit the experimental data, (Figure 7). If the diffusion region is next to the inhibitor film/metal interface, the mass transfer of reactive species is delayed, resulting in a diffusion characteristic of EIS, like Warburg impedance *W* [59]. The results of fitting the data to model C is presented in Table 3. Unlike the result of fitting of model B, the % efficiency of corrosion inhibition increases smoothly from 60 to 93% for the increase of Harmal extract concentration from 82.65 ppm to 826.5 ppm.

The increase in *R*_ct_ and decrease in CPE impedance values upon addition of Harmal extract indicated reduction in the corrosion rate with increase in electrical double layer thickness due to the formation of adsorbed protective film on the metal-solution interface. The inhibition action suppresses both the impedance Z_CPE_ and corrosion current density (*i*_corr_) due to replacement of water molecules present on the surface of steel electrode surface by the inhibitor ones [52], Table 1, Table 2 and Table 3.

The increase in the n value (0.905–0.997) in the case of inhibited system, compared to the n value obtained in the blank aggressive medium (0.800), could be attributed to a lessening of surface heterogeneities, Table 2 [60]. Moreover, a significant decrease in the electrical double layer capacitance Cdl values with increase in inhibitor concentration can be due to the development of a defensive film on the metal interface. The values of Cdl decrease from 229.0 μF/cm^2^ for the C-steel in the blank to 58.6 μF/cm^2^ for the optimum concentration of Harmal extract 283.4 ppm, then it remains almost constant, model B (Table 2). Meanwhile the values of *C*_dl_ decreases smoothly from 238.6 μF/cm^2^ for the blank to 36.74 μF/cm^2^ for the solution with 625.0 ppm Harmal extract, using model A fitting, Table 1.

The decrease in *C*dl can result from a decrease in local dielectric constant and/or an increase in the thickness of the electrical double layer, suggesting that inhibition by molecules can be assisted by their adsorption at the metal/solution interface due to increase in surface coverage (θ) [6,61]. The highest protection capacity values 98% occurred with 283.4 ppm the optimum concentration of added Harmal extract, Table 2.

**Figure 8:**
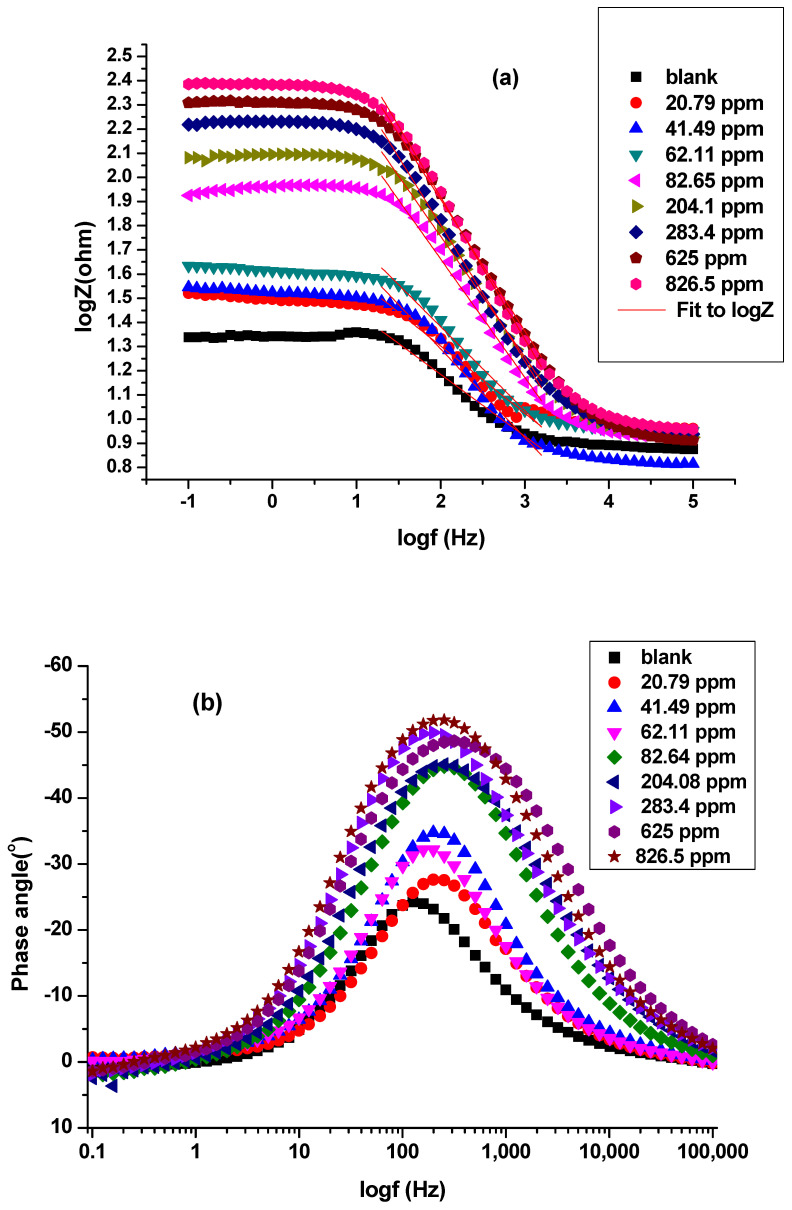
Bode (**a**) and Bode phase (**b**) plots of a C-steel electrode in 0.25 M H_2_SO_4_ solution with and without different concentrations of Harmal extract. The α values were calculated from the slopes (red lines) of Bode plots.

Bode and Bode phase plots: log (imaginary part of impedance) (log |Z| (ohm)), and phase angle (°) were plotted against log frequency (log*f* (Hz)), Figure 8a,b respectively, for C-steel in 0.25 M H_2_SO_4_ in the absence and presence of Harmal extract.

The α value indicates the alloy surface irregularity. It is calculated from the slope of the linear region of log(f) versus log|Z| plots (Figure 8a). The α values for C-steel in 0.25 M H_2_SO_4_ (with and without Harmal extract) were calculated. Ideally, the value of α should be equal to −1 for an ideal capacitor. Coarseness and heterogeneity of the C-steel surface was attained for less than −1 values. It can be seen that the presence of Harmal extract increased the α value to more negative values from −0.256, −0.279, −0.366, −0.347, −0.489, −0.499, −0.562, −0.543, to −0.587 for concentration of Harmal extract ranging from 0, 20.79, 41.49, 62.11, 82.65, 204.1, 283.4, 625.0, to 826.5 ppm. The decrease of heterogeneity of C-steel surface occurred as a result of adsorption of Harmal extract [61].

Figure 8b shows phase angle (°) plotted against (log *f* (Hz)). It is clear that increasing Harmal extract causes more negative values of phase angle at the intermediate frequency; the phase angle values were −24.15° (0 ppm), −32.43° (62.11 ppm), −44.44° (82.65 ppm), −44.93° (204.1 ppm), −49.89° (283.4 ppm), −48.91° (625 ppm), and −51.88° (826.5 ppm). As the concentration of Harmal extract increases, the values of phase angle become more negative. This indicates that the inhibitive behavior occurred because more of the Harmal molecules adsorbed at the C-steel surface [61].

### 3.3. Polarization Curve Measurements

**Figure 9:**
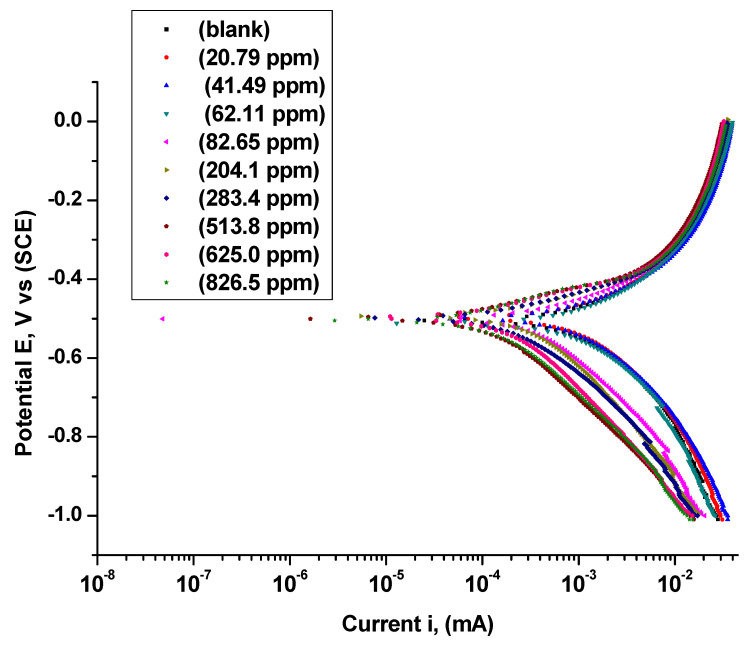
Tafel plots for C-steel in 0.25 M H_2_SO_4_ containing different concentrations of Harmal extract (0, 20.79, 41.49, 62.11, 82.65, 204.1, 283.4, 625.0 and 826.5 ppm).

The polarization curves for the corrosion of C-steel in 0.25 M H_2_SO_4_ in the presence and absence of Harmal extract were shown in Figure 9. The Tafel plots indicate that the addition of Harmal extract does change potential *E*_corr_ significantly, noting that the current density *i*_corr_ decreases, this is due to the inhibitory effect of the Harmal extract, Table 4. Furthermore, increasing the Harmal extract from 62.11 ppm to 826.5 ppm leads to alter cathodic Tafel slope βc (mV dec^−1^) much more than the anodic Tafel slope βa (mV dec^−1^) values. This indicates that more inhibition effect of Harmal extract occurs on the cathodic process than on the anodic process Table 4, [61].

Table 4 shows that the addition of the Harmal extract leads to a significant decrease in the corrosion current *i*_corr_ as it decreases from 12.70 mA/cm^2^ to 6.02 mA/cm^2^ in the absence and presence of 513.83 ppm of Harmal extract respectively. The corrosion potential *E*_corr_ shifts in a random way to more negative values with increasing Harmal extract concentration due to an increase in inhibition. Moreover, it is clear that a high IE% 50.2% was attained at 283.4 ppm of Harmal Extract, then it remains almost constant for further increase in concentration. This suggests that the Harmal extract has a moderate inhibition of C-steel corrosion based on polarization studies. Since the shift in *E*corr towards anode or cathode of inhibitor solutions with reference to blank solution is equal to 170 mV (greater than 85 mV), then the inhibitor is not a mixed inhibitor. It can be classified as a cathodic-type inhibitor [51].

There is a difference between the results of inhibition in EIS and potentiodynamic polarization techniques. Tafel analysis is based on current measurement in the time domain while electrochemical impedance in the frequency domain. Also, Tafel analysis is based on dc measurement, while EIS is on an ac one. A study has been performed to find how those two techniques can be correlated with less discrepancy between them, [62]. Here, the Tafel slopes (Tafel constants) are not well as values compared to the one obtained by impedance spectroscopy, particularly for less ideal Tafel plots like the ones we obtained. Thus, we adopt the θ values obtained from impedance techniques.

### 3.4. Comparison of IE%

The inhibition efficiency of Harmal leaves extract in sulfuric acid media was compared with those of three other plant extracts reported in the literature, Table 5. The results showed that Harmal extract is more effective toward inhibition of C-steel corrosion compared to leaves extract of Psidium Guajava, Tephrosia purpurea and Aegle marmelos, [22,63,64]. IE% for 204.1 ppm Harmal extract was 96.9%, while it was for 200 ppm extract of each of Psidium Guajava (49%), Tephrosia purpurea (89.4%), and Aegle marmelos (72.21%). Thus, Harmal extract was the most efficient inhibitor among the compared samples.

### 3.5. Inhibition Mechanism and Adsorption Isotherm

It is well established that the effect of the inhibitor on the corrosion rate is related to the adsorption of the anticorrosion molecules on the surface of the metal. The inhibition occurred by two mechanisms [65].

The first mechanism is geometric blocking, which depends on reduction reducing the surface area exposed to corrosion by adsorption of the anti-corrosion molecules. The second mechanism includes energy changing of the cathodic or anode reaction, which is called the energy effect. There is no procedure that can be used to specify which of the mechanisms is responsible for these processes. Theoretically, there is no change in the corrosion potential if the geometric blocking is stronger than the energy effect [66].

The inhibition effect can be explained by adsorption of Harmal extract at the C-steel surface. The Harmal extract replaces the water molecules at the metal interface according to the following Equation (5) [67,68]:

Harmal (sol) + nH_2_O(ads) → Harmal (ads) + nH_2_O(sol) (5)

**Figure 10:**
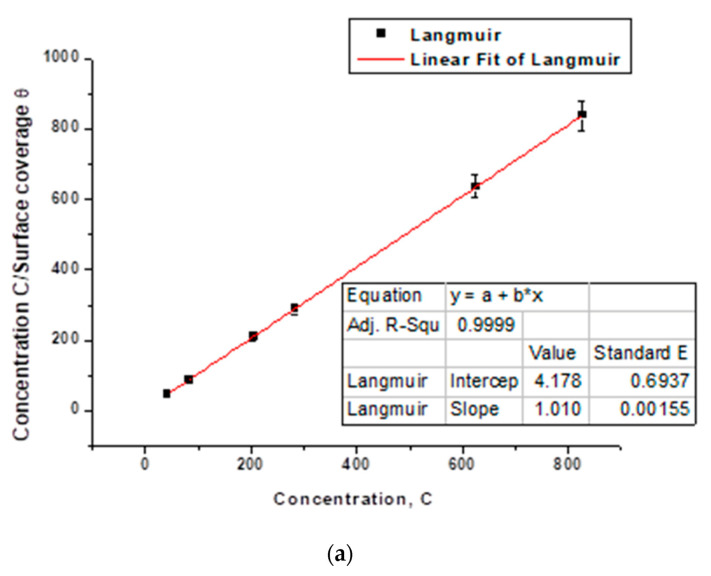

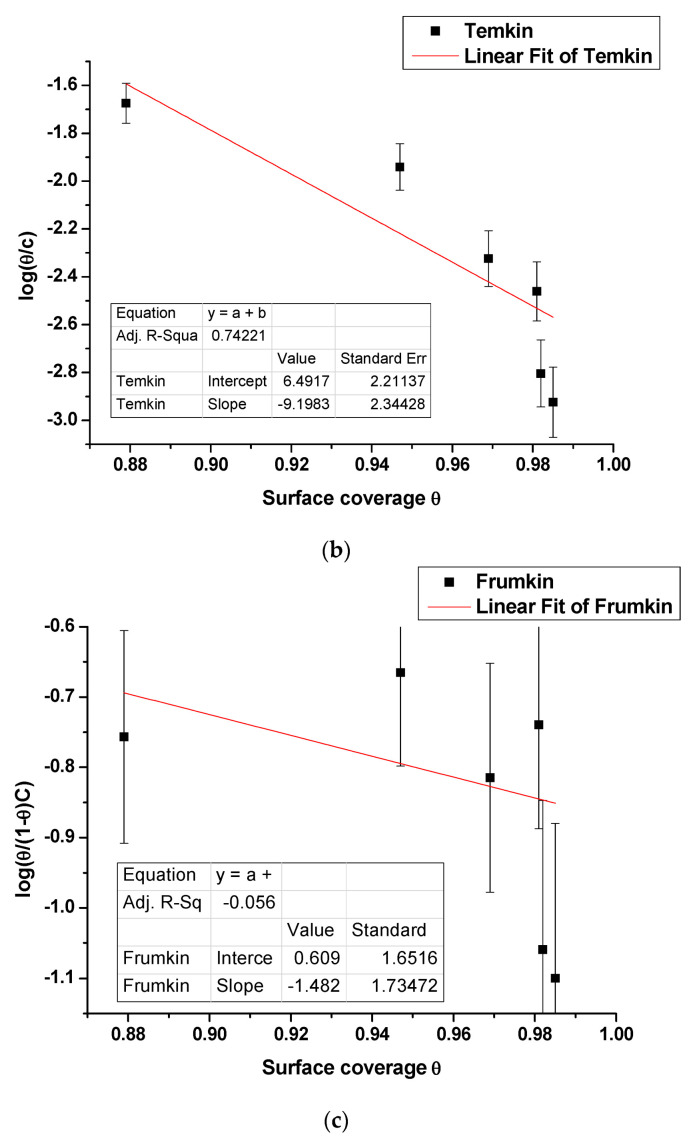
Adsorption isotherm from EIS plots for Harmal extract concentrations (ppm) on C-steel in 0.25 M H_2_SO_4_ medium: (**a**) Langmuir model, (**b**) Temkin model, and (**c**) Frumkin model.

For the fitting, the Langmuir adsorption model was applied. Plotting the experimental data C/θ versus C resulted in a fitted straight line as shown in Figure 10a. C (ppm) is the inhibition concentration of Harmal extract and θ is the surface coverage. It is clear that the adsorption follows Langmuir adsorption isotherm, as indicated by the adjusted correlation coefficient = 1.0 and the slope = 1.010 as expected from Langmuir model Equation (6) [61]:

c/θ = c + 1/*K*_ads_ (6)

Thus, the adsorption of Harmal extract as corrosion inhibitor was harmonious with Langmuir adsorption isotherm. The strength and stability of the adsorbed layer formed by Harmal extract was evaluated from inverse of the plot intercept. The *K*_ads_ was found to be equal to 0.239 (ppm^−1^).

The fitting of Temkin model was achieved by plotting log(θ/C) versus θ, Figure 10b. The obtained straight line has adjustable correlation coefficient R = 0.742. Thus, Temkin model is less acceptable than Langmuir model because of less R value [52].

The plot of Freumkin model, log(θ/(1−θ)C) versus θ gave a large error and almost zero R value, Figure 10c. Thus, this model is not suitable to explain adsorption of Harmal extract onto C-steel [52].

### 3.6. Surface Analysis

**Figure 11:**
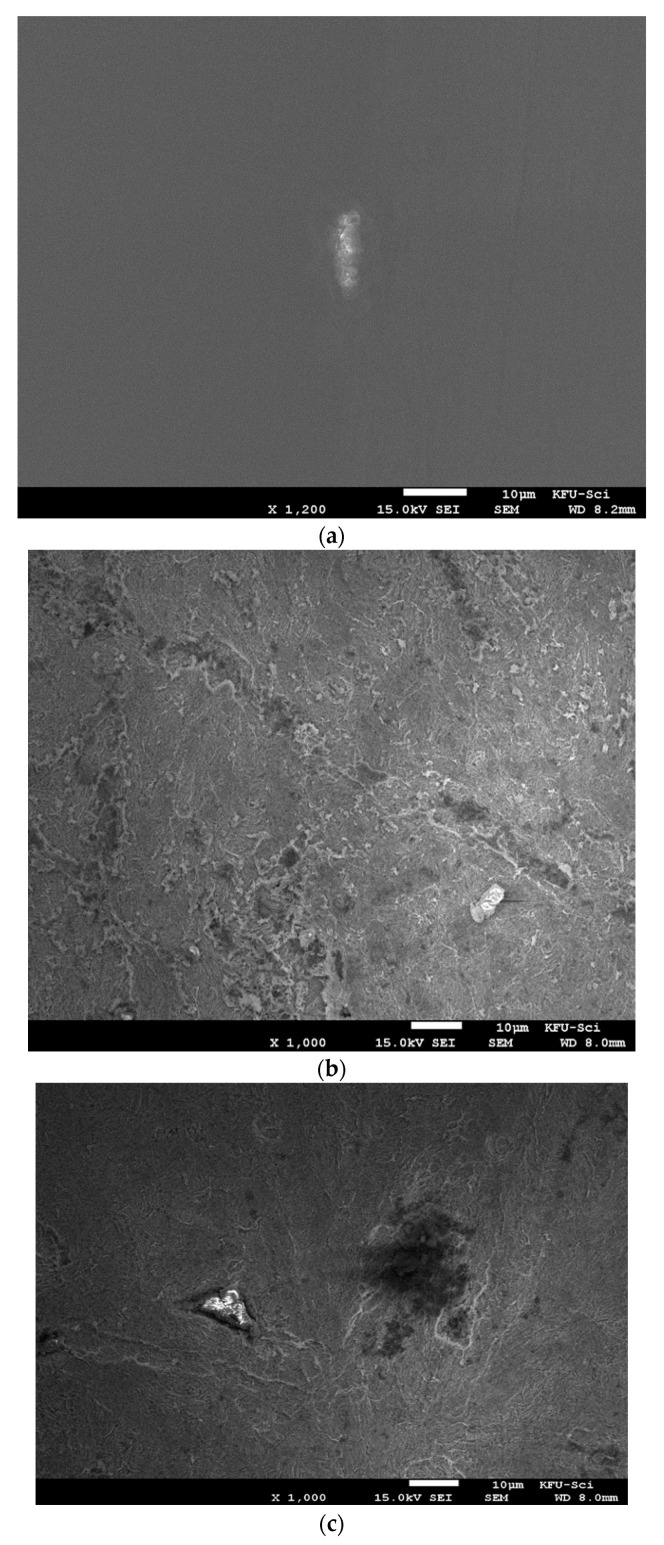
SEM micrographs for pristine C-steel (**a**) and C-steel immersed in 0.025 M H_2_SO_4_ for 3 h: corroded C-steel (**b**) and inhibited C-steel by addition of Harmal extract (332 ppm) (**c**).

SEM analysis was used to study microscopic surface morphology of pristine C-steel (Figure 11a) and C-steel immersed in H_2_SO_4_ solution (0.25 M) for 3 h without and with Harmal extract addition, Figure 11b,c respectively. The morphology in Figure 11b shows that the surface of C-steel without Harmal extract is rough and highly corroded. However, in the presence of 332 ppm Harmal extract (Figure 11c), the corrosion activity was suppressed, and a slightly smooth surface was observed. This can be related to the adsorption of Harmal extract on C-steel surface, which forms a monolayer of protection against corrosion activity [69].

## 4. Conclusions

In this work, the efficacy of Harmal extract as an environmentally friendly inhibitor was demonstrated by investigating the electrochemical behavior of C-steel in solutions of 0.25 M H_2_SO_4_. The polarization curves indicate that Harmal extract acts as a cathodic-type inhibitor. It was also observed from the impedance plots that the charge transfer process mainly controls the corrosion of C-steel. The electrical double layer capacitance values *C*_dl_ decreases while the charge transfer resistance *R*_ct_ increases with increase in Harmal extract concentration, indicating the formation of protective film. Moreover, Harmal extract demonstrates high efficacy of 98% using electrochemical impedance spectroscopy measurements at the optimum concentration 283.4 ppm. This indicates a high sensitivity of Harmal extract toward inhibition of C-steel in acidic medium. It was also observed that adsorption follows Langmuir isotherm. The electron-rich functional groups in the constituent of Harmal extract that contribute to anticorrosion, were determined by FTIR. SEM measurement of the steel electrode surface clearly demonstrates the inhibition effect of Harmal leaves.

## Figures and Tables

**Table 1:** Impedance parameters for Harmal extract on C-steel in 0.25 M H_2_SO_4_ medium by fitting the equivalent circuit model A.

Harmal Extract ppm	*R*_S_ Ω cm^2^	*R*_ct_ Ω cm^2^	Z_CPE_ µΩ^−1^ s^n^ cm^−2^	n	*C*_dl_ μF/cm^2^	θ	IE%
0	15.43	7.37 ± 1	519.2	0.877	238.6	-	-
20.79	18.26	11.18 ± 2	396.5	0.851	153.5	0.340	34.0
41.49	13.18	13.63 ± 2	356.9	0.852	141.6	0.459	45.9
62.11	17.88	16.37 ± 2	328.0	0.846	126.6	0.549	54.9
82.65	16.87	42.16 ± 4	144.3	0.840	54.74	0.825	82.5
204.1	16.90	59.32 ± 5	164.5	0.788	47.32	0.876	87.6
283.4	17.54	83.40 ± 7	148.2	0.804	50.77	0.912	91.2
625.0	15.98	102.25 ± 10	134.4	0.768	36.74	0.928	92.8
826.5	18.14	121.20 ± 13	118.8	0.796	40.00	0.939	93.9

**Table 2:** Impedance parameters for Harmal extract on C-steel in 0.25 M H_2_SO_4_ medium by fitting the equivalent circuit model B.

Harmal Extract ppm	*R*_S_ Ω cm^2^	*R*_f_ Ω cm^2^	Z_CPEf_ µΩ^−1^s ^n^ cm^−2^	n_f_	*R*_ct_ Ω cm^2^	Z_CPE_ µΩ^−1^s ^n^ cm^−2^	n	*R*_f_ + *R*_ct_	*C*_dl_ μF/cm^2^	θ	IE%
0	3.83	5.98	359.06	1.00	1.37 ± 1	1150.79	0.800	7.35	229.01	-	-
20.79	4.29	10.00	292.22	0.906	-	-	0.145	-	-	-	-
41.49	3.21	3.10	49,090	0.409	11.31 ± 2	248.46	0.926	14.41	155.36	0.879	87.9
62.11	4.53	16.11	311.12	0.853	0.68 ± 1	-	1.00	16.78	-	-	-
82.65	4.23	15.81	274.72	0.795	25.71 ± 3	122.50	0.996	41.52	120.35	0.947	94.7
204.1	4.16	14.00	460.91	0.708	44.03 ± 4	108.33	0.937	58.03	76.15	0.969	96.9
283.4	4.21	7.96	1269.35	0.625	73.70 ± 8	98.33	0.904	81.65	58.58	0.981	98.1
625.0	4.00	23.94	268.35	0.727	75.61 ± 7	96.16	0.915	99.54	60.87	0.982	98.2
826.5	4.48	29.27	418.94	0.704	89.38 ± 9	94.89	0.916	118.65	61.27	0.985	98.5

**Table 3:** Impedance parameters for Harmal extract on C-steel in 0.25 M H_2_SO_4_ medium by fitting the equivalent circuit Model C.

Harmal Extract ppm	*R*_S_ Ω cm^2^	*R*_f_ Ω cm^2^	Z_CPEf_ µΩ^−1^s ^n^ cm^−2^	n_f_	*R*_ct_ Ω cm^2^	Z_CPE_ µΩ^−1^ s ^n^ cm^−2^	n	*W* mS s^1/2^	*C*_dl_ μF/cm^2^	θ	IE%
0	3.78	0.60	4207.3	1.00	6.82 ± 1	308.7	1.00	11.47	308.73	-	-
20.79	4.51	7.83	221.2	0.975	3.97 ± 1	-	0.358	8.858	-	-	-
41.49	3.24	10.38	234.3	0.946	3.80 ± 1	-	0.999	9.442	-	-	-
62.11	4.41	13.01	236.9	0.924	3.87 ± 1	-	0.969	8.784	-	-	-
82.65	4.11	24.63	140.0	1.00	16.91 ± 2	113.0	0.931	4.915	71.08	0.597	59.7
204.1	4.07	40.15	115.0	0.957	17.80 ± 2	214.8	0.828	3.623	67.59	0.617	61.7
283.4	4.09	58.11	115.4	0.944	23.27 ± 3	194.4	0.907	2.310	111.74	0.707	70.7
625.0	3.94	12.59	226.0	0.786	86.87 ± 8	82.6	0.916	4.479	52.52	0.922	92.2
826.5	4.44	24.76	389.3	0.731	93.75 ± 9	89.2	0.925	7.970	60.53	0.927	92.7

**Table 4:** Polarization parameters for Harmal extract on C-steel in 0.25 M H_2_SO_4_ medium.

Harmal Extract ppm	*i*_cor_ mA cm^−2^	*E*_cor_ (V) vs. SCE	β_c_ mV dec^−1^	β_a_ mV dec^−1^	θ	IE%
0	12.70	−642.9	−580.9	794.4	0.000	0.0
20.79	12.60	−583.3	−610.5	712.8	0.008	0.8
41.19	12.41	−607.1	−474.1	690.7	0.023	2.3
62.11	10.00	−666.7	−480.7	705.1	0.213	21.3
82.65	9.06	−761.9	−423.1	808.1	0.286	28.6
204.10	8.12	−785.7	−390.8	791.1	0.361	36.1
283.40	6.32	−738.0	−364.1	680.2	0.502	50.2
513.83	6.02	−785.7	−294.1	758.1	0.526	52.6
625.00	6.65	−812.5	−309.7	791.8	0.477	47.7
826.50	6.45	−785.7	−340.9	748.5	0.493	49.3

**Table 5:** Percent inhibition efficiency (IE%) toward C-steel corrosion by extract of Harmal, Psidium Guajava, Tephrosia purpurea and Aegle marmelos leaves at various concentration C (ppm).

C_Harmal_ (ppm) This Work	IE%	C_Psidium Guajava_ (ppm) [22]	IE%	C_Tephrosia_ _purpurea_ (ppm) [63]	IE%	C_Aegle marmelos_ (ppm) [64]	IE%
0	-	0	-	0		0	
20.79	-	20	15	50	56.3	100	68.05
41.49	87.9	50	21	100	65.2	200	72.21
62.11	-	200	49	150	77.7	300	74.45
82.65	94.7	400	80	200	89.4	400	76.69
204.1	96.9	800	82	250	91.3	500	81.85
283.4	98.1	1200	82	300	92.5	-	-
625.0	98.2	-	-	350	91.1	-	-
826.5	98.5	-	-	400	90.9	-	-

## Sample Availability

Samples of the compounds are available from the authors.
